# Pipeline for generating stable large genomic deletions in zebrafish, from small domains to whole gene excisions

**DOI:** 10.1093/g3journal/jkab321

**Published:** 2021-09-09

**Authors:** Alisha Tromp, Kate Robinson, Thomas E Hall, Bryan Mowry, Jean Giacomotto

**Affiliations:** 1 Queensland Brain Institute, The University of Queensland, St Lucia, Queensland 4072, Australia; 2 Institute for Molecular Bioscience, The University of Queensland, St Lucia, Queensland 4072, Australia; 3 Queensland Centre for Mental Health Research, West Moreton Hospital and Health Service, Wacol, QLD 4076 Australia

**Keywords:** CRISPR, large, deletion, non-coding, indel, zebrafish, genomic region, guidelines, genetic compensation, heteroduplex

## Abstract

Here we describe a short feasibility study and methodological framework for the production of stable, CRISPR/Cas9-based, large genomic deletions in zebrafish, ranging from several base pairs (bp) to hundreds of kilobases (kb). Using a cocktail of four single guide RNAs (sgRNAs) targeting a single genomic region mixed with a marker-sgRNA against the pigmentation gene *tyrosinase*, we demonstrate that one can easily and accurately excise genomic regions such as promoters, protein domains, specific exons, or whole genes. We exemplify this technique with a complex gene family, neurexins, composed of three duplicated genes with multiple promoters and intricate splicing processes leading to thousands of isoforms. We precisely deleted small regions such as their transmembrane domains (150 bp deletion in average) to their entire genomic locus (300 kb deletion for *nrxn1a* for instance). We find that both the concentration and ratio of Cas9/sgRNAs are critical for the successful generation of these large deletions and, interestingly, that in our study, their transmission frequency does not seem to decrease with increasing distance between sgRNA target sites. Considering the growing reports and debate about genetically compensated small indel mutants, the use of large-deletion approaches is likely to be widely adopted in studies of gene function. This strategy will also be key to the study of non-coding genomic regions. Note that we are also describing here a custom method to produce the sgRNAs, which proved to be faster and more robust than the ones traditionally used in the community to date.

## Introduction

**Figure 1:**
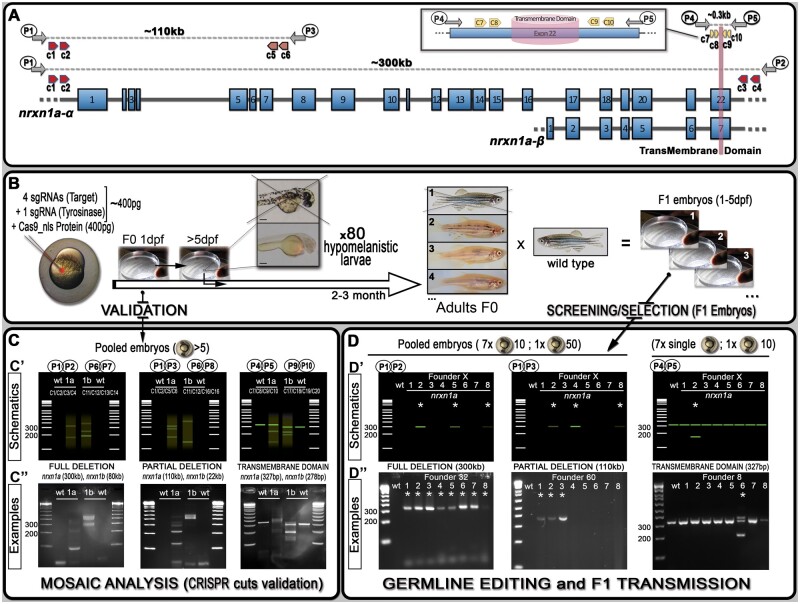
Schematics of neurexin organization and pipeline for generating mutants with large deletions. (A) Representation of neurexin1a (*nrxn1a*) genomic region together with sgRNA (CRISPR) target sites (c1–10) as well as primers used for validation and screening. The size of exons, intron gaps, and protein domains (and compared with one another) are not drawn to scale. (B) Pipeline designed to generate large deletions in zebrafish; 24 h post-injection, pools of five embryos are collected to evaluate the efficiency of the Cas9/sgRNAs mixes (see (C) mosaic analysis). Following validation, 80× 5dpf-larvae are selected based on their pigmentation phenotype. Once adult, F0 animals are crossed against wild-type to identify carriers/founders able to transmit the designed deletion to F1 mutants (see (D) stable validation). (C) To analyze/validate the efficiency of our sgRNAs-mixes, we proceeded with a basic PCR approach. Depending on the size of the target, a wild-type amplicon may be amplified; with the presence of a wild-type product hampering the amplification/detection of DNA remodeling (especially if rare). If large DNA deletion(s) happens, one should observe smearing with the presence of clear amplicon(s), as presented in the C’ schematics. The lower panel (C”) present the results we obtained for *nrxn1a* and *nrxn1b* transient validations. These transient validations can also be performed on individual embryos. (D) The same PCR approach is conducted to identify F0 adult founders. This time, eight batches are collected as schematically described. As opposed to the mosaic analysis (transient approach), a sharp band(s) should be observed, evidencing the presence of a large deletion. Upper panels (D’) represent theoretical results for one specific founder, while lower panels (D”) present PCR profiles obtained during our screen against the different *nrxn1a* deletion design. Founder frequency was evaluated at approximately 1/10. Asterisks* highlight positive founders carrying the desired mutation. All sequencing data as well as alignments and annotated gene files are available in the Supplementary zip file S04 with mutations summarized in Supplementary Table S04. A step-by-step protocol is also available in Supplementary file S05.

CRISPR/Cas9 has recently revolutionized genetics and the way we can manipulate gene function (Jinek *et al.* 2012). Commonly, to disrupt a gene using this technology, one employs a single guide/site strategy to promote targeted small indels [addition or deletion of 1–20 base pairs (bp)] inducing a frameshift and the generation of a premature stop codon. Although this strategy is straightforward and very efficient, there is growing literature reporting genetic compensatory mechanisms, *i.e.* the mutation is rescued/compensated by diverse mechanisms that are not yet fully understood (for details see Rossi *et al.* 2015; Anderson *et al.* 2017; El-Brolosy and Stainier 2017; Sztal *et al.* 2018; El-Brolosy *et al.* 2019; Buglo *et al.* 2020). Recently, we began to study the neurexin gene family in the zebrafish (Tromp *et al.* 2021). We experienced some challenges knocking out these genes using this traditional approach. Despite successfully generating small indels triggering a premature stop in the gene-open reading frames, we were still detecting expression of full-length proteins, suggesting exon skipping or use of alternative/cryptic splicing sites in all our mutants. When one has a close look at this gene family, it seems evident that these are perfect candidates for escaping traditionally engineered mutations. Indeed, these genes, very well conserved across species, display an extreme transcriptomic complexity that makes them very prone to genetic compensatory mechanisms (Treutlein *et al.* 2014). First, each gene presents two distinct promoters driving two major isoforms (α and β, Figure 1A), and most exons of each form can be alternatively spliced, leading to the expression of thousands of isoforms (Treutlein *et al.* 2014). Second, all vertebrates present at least three neurexin genes (duplicated in zebrafish) with high sequence identity, making them prone to cis-regulation via non-sense-mediated decay of one of the homologs (*i.e*. premature stop in *NRXN1* may trigger non-sense-mediated decay and up-regulation of *NRXN2* and/or *NRXN3*). To avoid, but also study, genetic compensation of these genes, we endeavored to establish mutants presenting (1) a full deletion of each entire gene, (2) a specific deletion of the long α-isoforms, and (3) deletion of shared domains such as the transmembrane region, essential for anchoring those proteins to the synaptic membrane. Considering the potential difficulty of these tasks (three large genes duplicated in zebrafish—six genes in total) and the absence of technical feasibility for removing hundreds of kilobases (kb), we first validated the possibility of generating such large deletions in the zebrafish.

Here, we not only report that it is feasible to accurately delete large genomic regions (ranging from several kb to potentially more than 1 Mb), but we also describe a pipeline to ease the selection of F0-founders (Figure 1). Such an approach has the potential to help generate mutants to better target/study small gene regions such as binding domains, catalytic sites, localization signals, and/or specific exons. The ability to accurately generate large deletions will also be very useful to study non-coding regions as well as enhancers and promoters. Finally, the generation of such mutants should also help the study of genes that are prone to genetic compensation as well as the study of those mechanisms *per se*.

## Methods

### Zebrafish maintenance

Adult zebrafish and embryos were maintained by standard protocols approved by the University of Queensland Animal Ethics Committee. Ethic approval AE213_18/AE213_18. Wild-type lines used in these studies are TAB background.

### Genomic target selection and single guide RNA full length oligo design

Genomic sequences targeted by our single guide RNAs (sgRNAs) have been selected using both CRISPRscan (https://www.crisprscan.org/) and CHOPCHOP (https://chopchop.cbu.uib.no/) websites based on the zebrafish genome annotation (GRCZ11). Several methods are available online for producing sgRNAs to inject; however, we experienced two main problems: (1) inconsistent, low RNA yields with the template extension method and (2) long turnover times with the plasmid cloning/PCR method. This led us to optimize our own method resulting in the production of high yield and quality sgRNAs. Based on the target selected in either CRISPRscan or CHOPCHOP, we copied this region (with mutation if necessary) into a full-length oligonucleotide template (Supplementary Figure/File S03) which includes a T7 promoter in 5′ and the necessary Cas9 sequence in 3′. We then ordered sense (top) and antisense (bottom) full length oligos used in the “sgRNA preparation” presented below.

### sgRNA preparation and storage

Oligonucleotides (top and bottom oligos) were produced by Macrogen, Inc. (South Korea, Supplementary Table S01) and reconstituted with Invitrogen Ultra-Pure Distilled water to 200 µM. Double strand DNA templates for sgRNA synthesis were generated by annealing top and bottom oligos together with NEB buffer 2.1 in Thermal Cycler (Bio-Rad C1000 Touch) at 95°C for 5 min, with lid temperature at 105°C. Once the cycle was complete, tubes were left in Thermal Cycler, with lid closed, for 1 h to allow slow temperature ramp down. The resulting template was purified using either Scientifix NucleoSpin Gel and PCR Clean-up Kit (#740609) or Invitrogen Gel Extraction and PCR Purification Kit (# K220001) according to manufacturer’s instructions, and eluted in 15 μL Invitrogen Ultra-Pure Distilled water. We further quantified the purified DNA using Nanodrop (ThermoFisher ND-1000). Next sgRNAs were transcribed using a quarter reaction of the Ambion MEGAshortscript T7 Transcription Kit (#AM1354) with a DNA template concentration of 400–800 ng and overnight incubation at 37°C. DNA templates were removed by addition of TURBO DNase at 37°C for 15 min. sgRNAs were then purified using Zymo Research RNA Clean & Concentrator Kit (R1015) and eluted in 15µL of Invitrogen Ultra-Pure Distilled water. Prior to storage at –80°C, we quantified the RNA using Nanodrop and checked RNA integrity on a 2% SB agarose gel.

### sgRNAs/Cas9 injection mix preparation

Each mix was prepared on ice the hour prior to injection. For calculating the amount to mix/pipette, we generated and used a template available in Supplementary Table S03. We used buffers and Cas9 protein from New England Biolabs (NEB, EnGen^®^ Spy Cas9 NLS, # M0646). We prepared a 10 µL mix that we stored on ice in a carrier box that was transported to the fish facility prior to setting up the injections.

### sgRNAs/Cas9 injections

One-cell stage or yolk injections were performed as previously described (Laird *et al.* 2016). Male and female adult TAB wild-type were separated the day before injection. On injection day, animals were mixed back together and monitored for mating behavior. Animals were left to mate for 30 min after the release of the first eggs. In the meantime, injections were calibrated using a microscope micrometer calibration ruler. Embryos were then collected and distributed on a Sylgard injection tray. Depending on the experiment, we injected 1 nl of Cas9/sgRNAs mix in either the yolk or the cell. Injected embryos were then collected and maintained in E3 medium supplemented with methylene blue. Embryos were screened when necessary as described in the manuscript, and the selected larvae were transferred to the fish facility nursery for monitoring and feeding from five days post fertilization (dpf).

### Genomic DNA extraction

Genomic DNA (gDNA) was extracted from embryos at 1 dpf. Freshly prepared DNA extraction buffer (1 M KCl, 0.5 M Tris pH 8, 1 M EDTA, 20% IGEPAL, 10% Tween-20) with Proteinase K (10 mg/mL) was added to embryo/s and digested in Thermal Cycler (Bio-Rad C1000 Touch) on the following program: 55°C for 2 h and 98°C for 10 min. gDNA was stored at –20°C. As described in the manuscript, for deletions involving the amplification of a wild-type band (transmembrane regions, Figure 1), gDNA was extracted from individual zebrafish embryos (*n* = 7) and a pool of 10 embryos (*n* = 1) for each F0/clutch screened. For deletions large enough to not involve the amplification of a wild-type band (whole gene or isoform-specific deletions), gDNA was extracted from 7× pool of 10 embryos and 1× pool of 50 embryos (whenever possible) for each F0/clutch screened.

### Validation via PCR amplification

sgRNAs/Cas9 cutting efficiency was evaluated by PCR amplification using primers specified in Figure 1 and Supplementary Table S02. We used AmpliTaq DNA polymerase (#N8080172) and followed manufacturer’s procedures. Briefly, PCR master mix was prepared with 10× PCR buffer II, 25 mM MgCl_2_, 10 mM dNTP, 10 uM forward and reverse primers, and AmpliTaq DNA Polymerase (5U/µL); 1 µL of extracted DNA was added to the master mix in a 25 µL final reaction volume and incubated in a Thermal Cycler. Conditions of the PCR amplification were: 95°C (1 min), then 40 cycles at 95°C (30 s)/56°C (30 s)/72°C (20–30 s), and a final extension at 72°C for 1 min. PCR amplicons were revealed using gel electrophoresis using a 2% SB agarose.

### Sequencing and F1 generation

The amplified PCR products of samples identified as founders were sent for sequencing to Macrogen, Inc. (South Korea) using the same forward and reverse primers as the PCR screen. Sequencing results and mutations/deletions were processed using a combination of manual and automatic analysis. For the automatic analysis, we used the following websites (http://yosttools.genetics.utah.edu/PolyPeakParser/; http://crispid.gbiomed.kuleuven.be/); however, in most cases, those *in silico* approaches failed to help generate valuable information and did not lead to the identification of mutations. The best way to proceed was to manually read the chromatograms and define the two alleles on a trial-and-error approach (see Supplementary Figure S06 for a workflow example) or to gel extract the desired band for downstream sequencing. Once zebrafish founders were confirmed with sequencing, these were out-crossed to wild-type TAB and grown for generating heterozygotes that would be identified through fin clipping. All sequencing data as well as alignments and annotated gene files are available in the Supplementary zip file S04 with mutations summarized in Supplementary Table S04.

### Detailed protocol

A step-by-step protocol is available in Supplementary Figure S05.

## Results and discussion

### Validation of large deletion events in injected embryos

To test our ability to generate stable mutants with large genomic deletions, we initially designed a strategy based on four sgRNAs with two guides on each side of the sequence to be removed (schematic representation with *nrxn1a* gene in Figure 1A). In theory, several steps could be performed to validate the efficiency of each individual sgRNA, such as *in vitro*, High-resolution melting analysis (HRMA), and/or T7E1 assays (Samarut *et al.* 2016; Sentmanat *et al.* 2018; Mehravar *et al.* 2019). However, we demonstrate here that a simple PCR approach on a subset of the injected embryos is sufficient to visualize/validate proper deletion events. We initially injected four guides against different regions of *nrxn1a* and *nrxn1b*, aiming to delete: (1) the full genomic region (≈300 and 80 kb respectively), (2) the α-isoforms (≈100 and 20 kb respectively), and (3) the transmembrane domain (≈0.15 kb) (Figure 1A, Supplementary Table S01). We collected a mix of five embryos from each injection, extracted gDNA, and performed PCR amplification with respective primers as presented in Figure 1 and Supplementary Table S02. For full and isoform-specific deletions, although no wild-type amplicon could be detected (wt-target too large to amplify), different bands as well as clear smears were noticeable with the injected embryos of each targeted gene, demonstrating DNA remodeling and the presence of large deletions in at least some cells of the collected embryos (Figure 1, B and C). For the transmembrane domain, the picture is not as evident considering that a wild-type band could be amplified, thereby reducing the relevance of a PCR/gel approach to highlight non-abundant DNA remodeling/deletions. Nonetheless, we were still able to detect smears and/or smaller bands than the wild-type amplicons in the injected embryos, again demonstrating the presence of significant deletions in our samples.

### Optimization of Cas9 and sgRNA concentration

**Figure 2:**
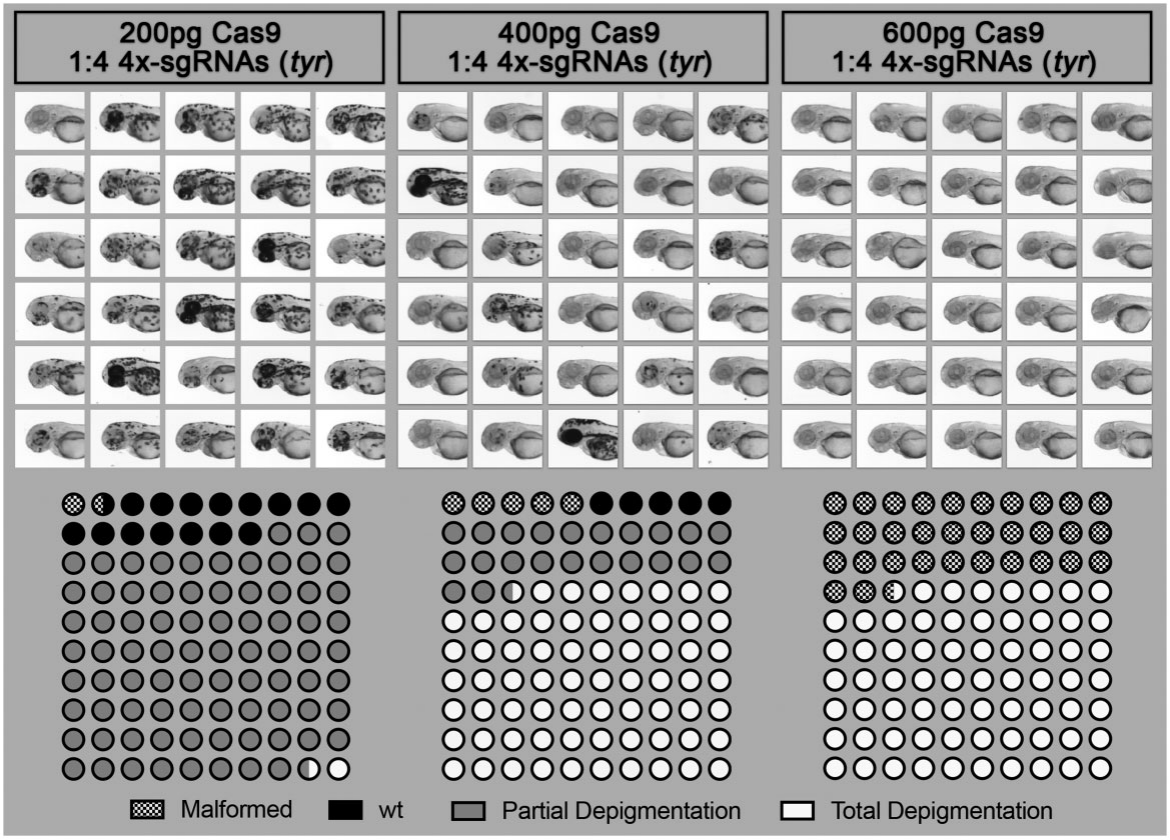
Effect of Cas9/sgRNA concentration on lethality, cutting efficiency, and phenotype outcome in zebrafish. Different concentrations of Cas9 protein mixed with 4× sgRNA guides against the gene tyrosinase (*tyr*) were injected into the yolk of one-cell stage zebrafish embryos. Injected animals were then observed at 2 dpf to score toxicity (malformation or death), partial depigmentation, or total depigmentation (suggesting complete bi-allelic *tyr*-knockout). A mix of 400 pg of Cas9/sgRNAs was selected to be used routinely in our pipeline.

Having demonstrated that we could visualize the presence of a large genomic deletion in our F0 samples, we next tested our ability to generate stable F1 lines (see below). At the same time, to improve the chance of successfully generating such mutants, we also focused on optimizing the concentration of sgRNAs and Cas9 to be injected. As a positive control, we targeted the pigmentation gene tyrosinase (*tyr*). We designed four different anti-*tyr* sgRNAs with target sites available in Supplementary Figure S01 (Jao *et al.* 2013). Although comparative studies across multiple genes and loci should be conducted to define optimal guidelines, we found that in our hands a concentration of 400 pg of Cas9 associated with a molar ratio of 1:4 sgRNAs represents an attractive compromise between toxicity and total absence of pigment in the observed larvae; evidencing bi-allelic knockout, and thereby high efficiency of the cuts (Figure 2, Supplementary Figure S02). Reducing the ratio of Cas9/sgRNA to 1:1 significantly reduced the efficiency as described below. We also compared yolk *vs.* blastomeric injection, which resulted in no clear difference in pigmentation loss. We therefore now perform our experiments with yolk-injection, facilitating the experimental procedure.

### Pipeline for generating heritable stable deletions

**Figure 3:**
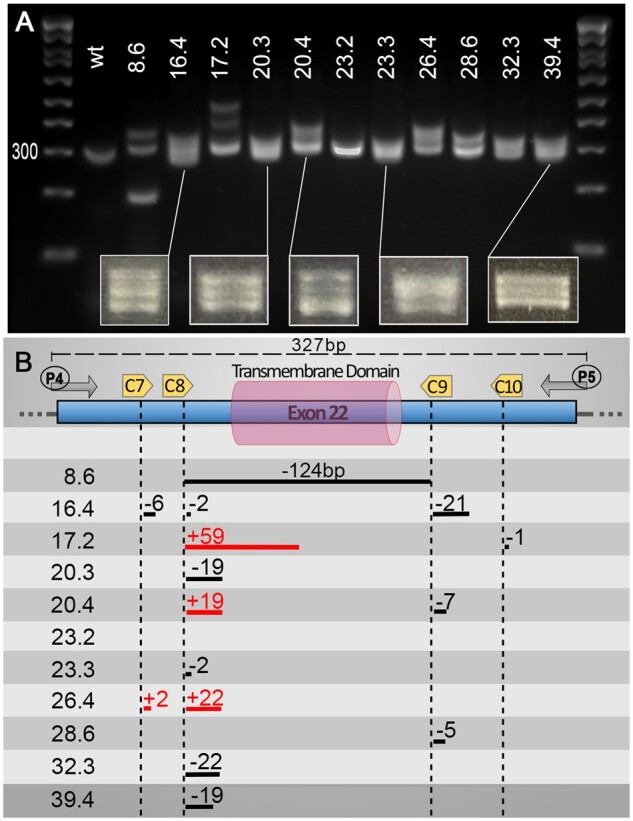
Heteroduplexes can help identify small indels using basic PCR screening approaches. Although not the primary aim of our pipeline, we found that, using a PCR screening approach, one can also easily detect “traditional” small indels due to an aberrant migration of heteroduplex PCR products. We found that at least a 2 bp difference is sufficient to trigger a significant slower migration of potential heteroduplexes. We found that the heteroduplexes were best observed with an amplicon size of around 300 bp, allowing clear visualization of two or three bands on the gels. Additionally, most of our targets/cuts did not sit in the center of the PCR amplicon, which might be a factor in promoting the formation of such heteroduplexes. (A) Gel presenting migration of PCR amplicons using primers P4 and P5 on validated F1 animals (Number X.x, *i.e.* 8.6, corresponds to fish number followed by embryo sample number). (B) Schematic representation of mutations found in the different animals presented in the upper panel. Distances are approximate only (not drawn to scale). Associated sequencing data are available in Supplementary zip file S07.

To test our ability to generate stable large deletions across multiple loci, we worked with a mix of five different guides, four against our target and one against *tyr* (tyr80, Supplementary Figure S01 and Table S01). We included an anti-*tyr* guide to help visualize correctly injected embryos and speed up the process of screening F0-founders (Figure 1B). At 5 days post-injection, we selected 80 injected larvae to grow based on their pigmentation phenotype, prioritizing those with greater pigment loss (Figure 1B and Supplementary Figure S02). Once grown to adulthood, we outcrossed those with wild-type to determine if any F0 fish would transmit a stable large deletion in F1 embryos (Figure 1B). As for the initial validation, it is straightforward to identify founders when the PCR reaction does not amplify a wt-amplicon as for total gene deletions. Therefore, only animals carrying such a deletion would lead to a PCR product on the gel, justifying the pooling of embryos/DNA from a large population to evaluate potential transmission (Figure 1D). However, for small deletions such as the transmembrane domain (≈0.15 kb), amplification of the wt-target could mask the signal from potential indels, especially in cases of low transmission. Based on these results, for screens that do not involve the amplification of wt-amplicon, we routinely collect eight pools (7 with 10 embryos plus an extra mix of up to 50 embryos) per dish to further extract gDNA and screen for potential deletions/mutants by PCR (Figure 1D). In contrast, for deletions involving the amplification of a wild-type band, we recommend not pooling the embryos, instead collecting seven individual embryos and only one pool of up to 10 embryos. Remarkably, we found at least one founder for each initially designed deletion. Importantly, we started our injections at a Cas9/sgRNAs molar ratio of 1:1 and obtained an average of 1 carrier/founder per 30 screened fish. Based on the results presented in Figure 2, we increased this ratio to 1:4 and observed an increase to approximately 1:10 founder. In total, we targeted the six *nrxn*-genes for the transmembrane domains (150 bp deletion on average) as well as *nrxn1a* and *nrxn1b* for the entire gene (300 and 70 kb deletion, respectively) and isoform-specific deletions (110 and 22 kb deletion, respectively). Although we anticipated that the larger the deletion, the harder it would be to isolate a mutant, this is not what we observed, with all deletions being obtained at a similar incidence. This contrasts with a recent study conducted by Wu *et al.*, who found that the frequency of this type of deletion decreases with increasing distance between sgRNA target sites (Wu *et al.* 2018). Finally, it is noteworthy that during the screens involving the deletion of the transmembrane domains, we found a high number of mutants presenting small indels at one or several sgRNA/target sites. Although the deletions or additions were relatively small, it was surprisingly easy to detect them on the gel facilitated by the formation of heteroduplexes. We were able to detect down to 2 bp differences as a result of the aberrant migration of the heteroduplex PCR products (Figure 3). This profile could be of great help to traditional approaches aiming to generate small indels (frameshift mutants). Although it is unclear what promoted such formation/migration, we hypothesized that the size (≈300 bp), as well as the position of the target sites (*i.e.* not in the middle of the amplicon), could play a role.

## Conclusion

Here, we demonstrated that large deletions ranging from hundreds of bp to hundreds of kb can be easily generated using multi sgRNA/target sites. Specifically, we found that the ratio of Cas9/sgRNAs has an effect on the founder rate, whereas the size/length of the deletion does not. To generate stable mutants, a concentration of 400 pg of Cas9 complexed with a molar ratio of 1/4 sgRNAs appears to offer an attractive approach, resulting in a final founder frequency of ≈ 1 in 10 injected fish. Supplementary Table S03 provides a template for the design and preparation of such cocktails. It is worth noting that this ratio and concentration are supported by a recent study aiming at establishing a method for generating null phenotypes in F0 zebrafish (Wu *et al.* 2018), although, for those “Morpholino-like” transient approaches, the authors found that 800 pg of Cas9 complexed with a 1:6 molar ratio was best for generating F0 null mutants. However, one must consider that the potential toxicity and phenotypic consequences of bi-allelic mutations could potentially hamper the chance to generate stable lines. Our findings also demonstrate that the simultaneous injection of a “marker” sgRNA/target, such as an anti-*tyr* guide, can greatly facilitate the selection of properly injected F0 embryos as well as aiding the selection of F0 adults with a greater chance of mutation transmission in F1 generations. The downside is that one must counter-select F1 and F2 animals free of *tyr*-mutations. Finally, as presented in detail in the methods, we experienced problems generating high/sufficient yield for our guides using the various methods found in the literature. This led us to design an alternative approach based on full length oligonucleotides. This proved slightly more expensive but yielded consistently large amounts of good quality sgRNAs.

## Data availability

All presented reagents are available upon request. The authors affirm that all data necessary for confirming the conclusions of the article are present within the article, figures, and tables. Supplementary material is available at figshare: https://doi.org/10.25387/g3.15130656.
